# Monitoring of diverse enteric pathogens across environmental and host reservoirs with TaqMan array cards and standard qPCR: a methodological comparison study

**DOI:** 10.1016/S2542-5196(21)00051-6

**Published:** 2021-05-05

**Authors:** Rachael Lappan, Rebekah Henry, Steven L Chown, Stephen P Luby, Ellen E Higginson, Lamiya Bata, Thanavit Jirapanjawat, Christelle Schang, John J Openshaw, Joanne O'Toole, Audrie Lin, Autiko Tela, Amelia Turagabeci, Tony H F Wong, Matthew A French, Rebekah R Brown, Karin Leder, Chris Greening, David McCarthy

**Affiliations:** a Department of Microbiology, Biomedicine Discovery Institute, Monash University, Clayton, VIC, Australia; b School of Biological Sciences, Monash University, Clayton, VIC, Australia; c Department of Civil Engineering, Monash University, Clayton, VIC, Australia; d School of Public Health and Preventive Medicine, Monash University, Clayton, VIC, Australia; e Water Sensitive Cities Institute, Monash University, Clayton, VIC, Australia; f Monash Sustainable Development Institute, Monash University, Clayton, VIC, Australia; g Division of Infectious Diseases and Geographic Medicine, Stanford University, Stanford, CA, USA; h Cambridge Institute for Therapeutic Immunology and Infectious Disease, University of Cambridge, Cambridge, UK; i Division of Epidemiology and Biostatistics, School of Public Health, University of California Berkeley, CA, USA; j School of Public Health, Fiji National University, Suva, Fiji

## Abstract

**Background:**

Multiple bacteria, viruses, protists, and helminths cause enteric infections that greatly impact human health and wellbeing. These enteropathogens are transmited via several pathways through human, animal, and environmental reservoirs. Individual qPCR assays have been extensively used to detect enteropathogens within these types of samples, whereas the TaqMan array card (TAC), which allows simultaneous detection of multiple enteropathogens, has only previously been validated in human clinical samples.

**Methods:**

In this methodological comparison study, we compared the performance of a custom 48-singleplex TAC relative to standard qPCR. We established the sensitivity and specificity of each method for the detection of eight enteric targets, by using spiked samples with varying levels of PCR inhibition. We then tested the prevalence and abundance of pathogens in wastewater from Melbourne (Australia), and human, animal, and environmental samples from informal settlements in Suva, Fiji using both TAC and qPCR.

**Findings:**

Both methods exhibited similarly h specificity (TAC 100%, qPCR 94%), sensitivity (TAC 92%, qPCR 100%), and quantitation accuracy (TAC 91%, qPCR 99%) in non-inhibited sample matrices with spiked gene fragments. PCR inhibitors substantially affected detection via TAC, though this issue was alleviated by ten-fold sample dilution. Among samples from informal settlements, the two techniques performed similarly for detection (89% agreement) and quantitation (R^2^ 0·82) for the eight enteropathogen targets. The TAC additionally included 38 other enteric targets, enabling detection of diverse faecal pathogens and extensive environmental contamination that would be prohibitively labour intensive to assay by standard qPCR.

**Interpretation:**

The two techniques produced similar results across diverse sample types, with qPCR prioritising greater sensitivity and quantitation accuracy, and TAC trading small reductions in these for a cost-effective larger enteropathogen panel enabling a greater number of enteric pathogens to be analysed concurrently, which is beneficial given the abundance and variety of enteric pathogens in environments such as urban informal settlements. The ability to monitor multiple enteric pathogens across diverse reservoirs could allow better resolution of pathogen exposure pathways, and the design and monitoring of interventions to reduce pathogen load.

**Funding:**

Wellcome Trust Our Planet, Our Health programme.

## Introduction

Diarrhoeal disease due to inadequate sanitation and poor water quality is a major public health issue and a target of one of the UN Sustainable Development Goals (SDG 6). This problem disproportionately affects lower-income and middle-income countries, especially people living in urban informal settlements.^1^, ^2^ Approximately 500 000 children under the age of 5 years die from diarrhoeal disease each year,^3^, ^4^, ^5^ despite the potential to prevent an estimated 360 000 child deaths annually by improvements to water, sanitation, and hygiene (WASH).^6^ Various non-diarrhoeal pathogens, most notably helminths, also contribute to enteric disease burden and malnutrition.^7^ Moreover, asymptomatic or subclinical carriage of various enteropathogens also impacts child growth.^8^ Recent evidence has suggested that traditional household-level WASH interventions such as pit latrines, handwashing with soap, and chlorination of water deliver suboptimal reductions in enteric disease in environments that are densely populated,^9^ highly contaminated,^10^ or have a high prevalence of diarrhoea.^11^ This finding is probably due to the inability of these interventions to address the many pathways that connect environmental enteropathogens to community residents. Humans, animals, and their surrounding environments can serve as extensively interconnected reservoirs for enteropathogens. Thus, unified one health and planetary health approaches are needed to identify pathogen exposure pathways and inform interventions to reduce pathogen load in the environment and in turn reduce human exposure.^12^

**Research in context**

**Evidence before this study**

Enteric pathogens contribute substantially to the disease burden in urban informal settlements, where interaction with animals and inadequate water quality and sanitation facilitate complex pathogen transmission pathways. A common approach to monitoring enteropathogen contamination in the environment is the use of faecal indicator organisms as a proxy for human faecal contamination because it is prohibitively intensive to screen for the multiple viruses, bacteria, protists, and helminths that cause enteric infections via standard qPCR. However, these indicators do not correlate with all relevant pathogens, provide no information on specific pathogen contamination or load, and fail to capture the importance of animals in complex pathogen transmission routes. The TaqMan array card has been used in large multicentre studies to simultaneously detect and quantify a broad range (>30) of enteric pathogens in human faecal samples. However, its use has rarely been extended to detection in the animal and environmental reservoirs integral to many enteropathogen transmission pathways and to our knowledge, it has never been evaluated against standard qPCR for this purpose. We searched the Scopus and Google Scholar databases for studies applying TaqMan array cards for pathogen detection using terms including “TaqMan array card”, “TaqMan low density array”, “pathogen”, “soil”, “water”, “environmental”, and “multiple pathogen detection”. To the best of our knowledge, only two studies have used TAC to detect enteropathogens in environmental samples.

**Added value of this study**

Our study provides the first comprehensive comparison between the standard qPCR and TaqMan array card molecular techniques for the detection of enteric pathogens in humans, animals, and the environment. We analysed mock samples with spiked genetic material of known concentration to evaluate the two techniques independently. In doing so, we highlight that some sample matrices are challenging for pathogen detection irrespective of method; other method comparison studies commonly use qPCR detection as a gold standard against which another method is compared, facing the limitation that it could also perform poorly in some sample matrices. We show that TAC is similar to standard qPCR for the monitoring of enteropathogens across sample types from multiple reservoirs. Additionally, our analysis of 46 pathogen and faecal indicator targets in samples from informal settlements in Fiji provides insight into enteropathogen contamination in a previously uncharacterised environment.

**Implications of all the available evidence**

Our results indicate that although TAC is overall slightly less sensitive and accurate than standard qPCR, it performs similarly for the detection of enteropathogens in environmental samples. Our application of the broad TaqMan array card panel of more than 30 enteropathogens to child stool, animal scat, soil, and water samples from urban informal settlements in Fiji revealed a contaminated environment with high enteropathogen diversity, indicating the usefulness of this technique for one health and planetary health studies. TAC presents a higher resolution alternative to faecal indicator analysis and a convenient cost-effective method for the high-throughput simultaneous detection of a large number of pathogen targets in different host and environmental reservoirs.

Assessing the extent of enteropathogen contamination and the effect of new mitigating interventions in urban informal settlements requires methods that can monitor several enteropathogen species in a range of sample types. Screening for a large number of enteropathogens is important, as multiple viruses, bacteria, protists, and helminths can be responsible for poor gastrointestinal health and diarrhoeal disease,^13^, ^14^ and mixed infections are common.^14^ Additionally, the relative contribution of individual pathogens to disease burden varies across settlements, within a settlement over time, and between individuals. Interventions can also disrupt some transmission pathways more effectively than others.^10^ The use of a catch-all approach has traditionally not been attempted due to the challenge posed by large numbers of possible enteropathogens, with the use instead of simpler solutions relying on bacterial indicator organisms to identify faecal contamination.^15^, ^16^ However, faecal indicators do not correlate well with pathogen abundance and distribution,^17^, ^18^, ^19^, ^20^ and reliance on indicators misses the complexities of enteropathogen diversity and pathogen-specific effects of an intervention. Thus, the development of high-throughput molecular methods for enteropathogen screening of human, animal, and environmental samples would remove the need to rely solely on faecal indicators and can provide a comprehensive view of enteropathogen sources and diversity.

TaqMan qPCR is a standard technique used across the human, animal, and environmental health fields to detect and quantify pathogens on the basis of amplification of a pathogen-specific gene sequence.^19^, ^21^, ^22^, ^23^ This technique can be readily used to quantify pathogenic bacteria, viruses, protists, and helminths in situ, whereas alternative approaches such as selective cultivation, amplicon sequencing, and metagenomic sequencing are more challenging to implement for non-bacterial targets. Moreover, given the ability to multiplex qPCR reactions and to use 96-well and 384-well plates to process many samples at a time, this technique is relatively efficient, cheap, and high-throughput with respect to sample numbers. However, the price and labour time for screening many samples for several pathogens can become high, because each additional pathogen target adds to the cost of reagents, sample volume used, and preparation time. These costs can become prohibitive for enteropathogen detection across human, animal, and environmental samples, where the number and taxonomic diversity of enteropathogens contributing to the burden of disease may be high and is often unknown.^24^

The TaqMan array card (TAC) is a microfluidic card designed to automate several TaqMan qPCR assays per sample. Originally designed for gene expression experiments, TAC has been effectively repurposed for detection of large panels of pathogens,^25^ with successful application to human faecal,^26^ blood,^27^ cerebrospinal fluid,^28^ and nasopharyngeal^29^ samples. Generally, the ability to efficiently detect large numbers of pathogens simultaneously is accompanied by a loss of sensitivity compared with standard qPCR,^30^, ^31^ although it is highly cost-effective compared with standard qPCR for the breadth of targets that can be detected. Several large multicentre studies have used TAC to study the aetiology of diarrhoeal disease.^14^, ^32^ However, TAC has rarely been applied to non-human samples, with only two studies to date using TAC to detect enteropathogens in food^33^ and environmental^18^ samples. The latter study showed that TAC can detect a wide range of pathogens in soil and water samples from informal settlements in Kisumu, Kenya.^18^ However, the sensitivity, specificity, and quantitation accuracy of TAC has not yet been extensively evaluated in environmental samples in relation to the gold standard of qPCR. Thus, it is currently unclear whether the technique presents a valid alternative to standard qPCR to monitor multiple enteropathogens across different reservoirs.

In this study, we designed and evaluated a custom enteropathogen TAC that detects 46 different pathogen marker and faecal indicator genes. Through this approach, we aimed to test whether TAC enables the reliable monitoring of enteropathogens across environmental and host reservoirs.

## Methods

### Study design and participants

In this methodological comparison study, we first comprehensively tested the specificity, sensitivity, and accuracy of standard qPCR and TAC on a set of mock samples consisting of spiked enteropathogen genomic DNA in different sample matrices varying in PCR inhibition levels. We additionally tested both techniques on wastewater samples from Melbourne (VIC, Australia) and human stool, animal scat, environmental water, potable water, and soil samples from informal settlements in Suva, Fiji, where the diversity and prevalence of enteropathogens was not previously known.

Samples of child stool (n=60), animal scats (n=17), soil (n=24), potable water (n=10), and environmental water (n=10) were collected from informal settlements in Suva, Fiji, as part of the Revitalising Informal Settlements and their Environments programme (RISE), a transdisciplinary research programme and randomised controlled trial focused on improving environmental and human health in urban informal settlements of Fiji and Indonesia.^34^, ^35^ A total of 314 stool samples were collected from children younger than 5 years from 12 informal settlements during Sept 27 to Nov 8, 2019; 60 samples were then randomly selected using the base random number generator in R statistical software, ensuring inclusion of a minimum of four samples from each settlement. Water samples were collected in clean, source-water rinsed disposable bottles from the associated settlement and then filtered; potable water was collected from local municipal water sources, and environmental water samples were taken from riverine, freshwater, and stormwater sources. Soil samples and domestic animal scats (of dog, chicken, and duck origin, and one of undetermined origin) were collected around the 12 informal settlements in Fiji.

Wastewater samples from Melbourne were collected between 2015 and 2020 from the inlet channel, as well as the effluent of primary settling ponds, from the Eastern Treatment Plant (Bangholme, VIC, Australia).

Ethics review and approval was provided by participating university and local institutional review boards, including Monash University Human Research Ethics Committee (Melbourne, Australia; protocol 9396) and the Fiji National University College Health Research Ethics Committee (Suva, Fiji; protocol 137.19). In Fiji, all study settlements, households, and caregivers or respondents provided written informed consent.

### Randomisation and masking

Two sets of mock samples were prepared by spiking (ie, artificially contaminating) samples with synthetic gene blocks (Integrated DNA Technologies; Coralville, IA, USA) representing eight enteropathogen genes (table 1; appendix 1 p 6). Set 1 consisted of ten nuclease-free water samples for comparison of method sensitivity and specificity. Set 2 consisted of previously extracted Australian samples from different matrices to test the performance of the two methods under varying levels of PCR inhibition. Full details on these mock samples can be found in appendix 1 (p 2) and appendix 2. All spiked samples were double-blinded: laboratory staff performing the qPCR (RH) and TAC (RL) assays and data processing were not aware of the concentrations present in the samples; an independent laboratory staff member (CS) was in charge of spiking the samples, which were randomly renumbered by another investigator (DM); and two separate laboratories delivered the TAC and standard qPCR results.

**Table 1:** TaqMan qPCR assays used for detection by standard qPCR and custom TaqMan array cards

	**Targeted gene**	**Forward primer (5’ to 3’)**	**Reverse primer (5’ to 3’)**	TaqMan probe (standalone qPCR)*
*Campylobacter jejuni, C coli*	*cadF*	CTGCTAAACCATAGAAATAAAATTTCTCAC	CTTTGAAGGTAATTTAGATATGGATAATCG	5’HEX-CATTTTGACGATTTTTGGCTTGA-3’MGB
*Salmonella enterica*	*invA*	TCGGGCAATTCGTTATTGG	GATAAACTGGACCACGGTGACA	5’FAM-AAGACAACAAAACCCACCGC-3’MGB
STEC	*stx1*	ACTTCTCGACTGCAAAGACGTATG	ACAAATTATCCCCTGWGCCACTATC	5’Texas Red-CTCTGCAATAGGTACTCCA-3’MGB
STEC	*stx2*	CCACATCGGTGTCTGTTATTAACC	GGTCAAAACGCGCCTGATAG	5’FAM-TTGCTGTGGATATACGAGG-3’MGB
EPEC	*eae*	CATTGATCAGGATTTTTCTGGTGATA	CTCATGCGGAAATAGCCGTTA	5’FAM-ATACTGGCGAGACTATTTCAA-3’MGB
*Cryptosporidium*	18S rRNA	GGGTTGTATTTATTAGATAAAGAACCA	AGGCCAATACCCTACCGTCT	5’FAM-TGACATATCATTCAAGTTTCTGAC-3’MGB
*Giardia*	18S rRNA	GACGGCTCAGGACAACGGTT	TTGCCAGCGGTGTCCG	5’HEX-CCCGCGGCGGTCCCTGCTAG-3’MGB
*Bacteroides*	16S rRNA	ATCATGAGTTCACATGTCCG	CTTCCTCTCAGAACCCCTATCC	5’FAM-CTAATGGAACGCATCCC-3’MGB
Internal amplification control†	16S rRNA	ATCATGAGTTCACATGTCCG	CTTCCTCTCAGAACCCCTATCC	5’VIC-AACACGCCGTTGCTACA-3’MGB

The following assays were multiplexed: *cadF* and *invA*; *Giardia* and *Cryptosporidium*; *Bacteroides* and the internal amplification control; *stx1* and *stx2*. The *eae* assay was run singleplex. Prior to the use of multiplex assays, standard curves were generated for the singleplex and multiplex formats and evaluated to confirm the absence of cross-target amplification or inhibition. STEC=Shiga toxin-producing *Escherichia coli*. EPEC=enteropathogenic *Escherichia coli*.

* TaqMan array card probes were identical with the exception of the fluorophore (all TAC probes were 5’FAM 3’MGB).

† The internal amplification control targets *Bacteroides* and was applied to standalone qPCR assays only.

For set 1, three samples contained all eight targets at low (10 copies per μL), medium (100 copies per μL), or high (1000 copies per μL) concentrations; six samples contained random combinations of targets and concentrations; and one sample was a blank with no targets spiked. The six randomised samples were determined by allocating each target for each sample with a random integer between 1 and 3 (where 1 was classified as low concentration) using the RANDBETWEEN function in Microsoft Excel (Office 365 version 1908). Set 2 of mock samples (36 samples) included: nine wastewater samples with no gene blocks spiked; seven potable water samples spiked with 200 copies per μL of each target; and five different combinations of low, medium, and high spiked targets in extracted DNA from each of four additional matrices.

### Procedures

Total genomic DNA was isolated from child stool, animal scat, and soil samples using the QIAGEN DNeasy PowerSoil Pro kit (QIAGEN; Hilden, Germany). Water and wastewater samples were passed through 0·22 μm filters from which DNA was extracted with the QIAGEN DNeasy PowerMax Soil kit (QIAGEN; Hilden, Germany). Both kits involve a bead-beating step. At least one negative extraction control was included in each extraction batch. Full details on sample collection, storage and DNA extraction can be found in appendix 1 (p 2). Extracted nucleic acid samples were frozen at −80°C before ambient transfer and refrigeration upon receipt in Melbourne, Australia. Extracted nucleic acid from Melbourne's wastewater samples were frozen at −20°C and defrosted on the day of the analysis on ice before use.

Standard TaqMan qPCR assays were undertaken using primers and probes for eight bacterial and protist pathogens (table 1). These pathogens were chosen because they are abundant in faecally contaminated environments^9^, ^18^ and can be reliably quantified through previously optimised qPCR assays.^36^ Up to two targets were multiplexed under the PCR reaction and cycling conditions (40 cycles) described in the US Environmental Protection Agency Method 1696.^37^ The PCR was done with on a Biorad CFX96 thermocycler (Biorad, USA) using TaqMan Environmental Master Mix v2.0 (Applied Biosystems; Pleasanton, CA, USA). Standard curves were prepared using the gene blocks (appendix 1 p 6),^38^ and each reaction contained an internal amplification control gene block to indicate PCR inhibition. Quality control, data analysis and calculations were done as outlined in Method 1696 (appendix 1 p 3).^37^

The custom TAC (Applied Biosystems; Pleasanton, CA, USA) contained 48 singleplex assays (appendix 1 pp 5, 8), including the eight primer and probe sets used in the standard qPCR assays and the manufacturer's 18S rRNA control. Of the 47 custom assays, 40 have previously been validated on TAC^26^, ^36^ and the remaining seven were assays that have previously been published as individual TaqMan qPCR assays under similar conditions.^14^, ^39^, ^40^, ^41^ Cards were prepared as described by Liu and colleagues^26^ (see appendix 1 p 3 for details).

To calculate gene copies per μL, a standard curve was generated using synthetic plasmid controls (GeneWiz) as described in Kodani and Winchell^42^ (details in appendix 1 p 3). The plasmid insert sequences are in appendix 1 (p 11). This positive control was run in triplicate with a no-template control on each card. The lower limit of quantitation (LLOQ) was defined as the lowest dilution of the standard curve that was detectable in all three replicates (appendix 2).

### Statistical analysis

TAC data were reviewed within the QuantStudio Real-Time PCR Software v1.3. Each multicomponent plot for TAC data was manually checked for amplification, and quantification/threshold cycle (C_q_) values were checked and manually adjusted per target when the automatic threshold was inappropriate. Samples with very poor amplification curves were considered negative results. C_q_ values were used to calculate gene copies per μL of original nucleic acid extract using the standard curve for each target. For the purpose of this method comparison, all assays with a genuine amplification curve, regardless of C_q_ value, were included in the analysis.

Sensitivity and specificity for both methods were calculated using the spiked samples as follows:

$$\text{Sensitivity}=\frac{\text{true positives detected}}{\text{total spiked positives (ie, true positives}+\text{false negstives)}}$$

$$\text{Specificity}=\frac{\text{true negatives detected}}{\text{total non-spiked negatives (ie, true negatives}+\text{false positives)}}$$

To assess quantitation accuracy, the percentage of assays that quantified gene copies per μL within one log_10_ of the spiked amount was calculated as follows:

$$|\log_{10}(\text{spiked copies per}\mu \text{L})-\log_{10}(\text{measured copies per}\mu \text{L})|<1$$

For the second set of mock samples, specificity was not calculated because it was possible for spiked targets to already be present in the samples (false positives could not be identified). Additionally, samples with a background level of target detected by either method were excluded from the quantitation accuracy calculations.

All statistical analyses were done in R v3.6.2.^43^ Cohen's κ statistic was calculated to quantify agreement between standard qPCR and TAC sensitivity. Wilcoxon's signed-rank test was applied with continuity correction using the wilcox.test() function. R^2^ values for concordance between measured qPCR and TAC gene copy numbers were calculated with the lm() function using log_10_ transformed copy numbers with a pseudocount of 1 to accommodate values of 0. Cohen's κ statistic was calculated with the kappa2() function from package irr v.0.84.1.^44^ Graphics were created with ggplot2 v3.3.2.^45^

### Role of the funding source

The funders of the study had no role in data collection, analysis, interpretation, writing or the decision to submit the manuscript.

## Results

**Figure 1:**
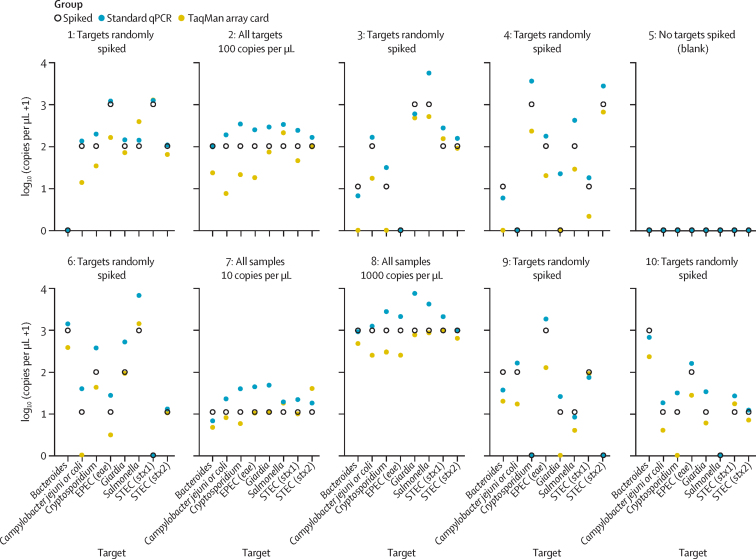
Quantitation of spiked genetic material in nuclease-free water by TAC and standard qPCR Ten different combinations of spiked material were tested in a randomised double-blinded manner. Figure shows samples spiked randomly in different combinations (samples 1, 3, 4, 6, 9, 10); those spiked at consistent concentrations of 10 copies per μL (sample 7), 100 copies per μL (sample 2), or 1000 copies per μL (sample 8); or not spiked at all (sample 5; a blank control). For each target, the quantity of material spiked (white circle), the copies detected by standard qPCR (blue circle), and the copies detected by TAC (yellow circle) are shown. TAC=TaqMan array card. EPEC=enteropathogenic *Escherichia coli*. STEC=Shiga toxin-producing *Escherichia coli*.

General assay sensitivity and specificity were tested using synthetic gene blocks of eight pathogen markers spiked at different concentrations into nuclease-free water in ten different combinations (80 individual assays; appendix 2). Across all assays (64 true positives and 16 true negatives), the sensitivity of TAC was slightly lower (92%, 59/64) than qPCR (100%, 64/64). TAC performed well when detecting all eight targets at low concentration (10 copies per μL), but sometimes failed to detect targets at this concentration when others were present at high concentration (1000 copies per μL; figure 1). Specificity was very high for both assays, with no false positives detected via TAC (100%, 16/16) and one false positive detected by qPCR (94%, 15/16). Both methods quantified spiked targets with variable accuracy in nuclease-free water, with TAC on average underestimating target abundance by 1·73-times qPCR by contrast overestimated target abundance by 2·61-times (figure 1; appendix 2). Overall, 79 (99%) of 80 qPCR results were within one log of the known concentration, compared with 73 (91%) of 80 for TAC (table 2). Five of the seven discrepant TAC results were instances of low-copy targets that were not detected (ie, ten copies per μL spiked, no copies detected—attributable to TAC's lower sensitivity).

**Table 2:** Performance of TAC and qPCR on spiked samples in sample matrices varying in levels of PCR inhibitors

	**Sensitivity**		**Accuracy**	
	TAC	qPCR	TAC	qPCR
**Sample matrix**				
Nuclease-free water	92% (59/64)	100% (64/64)	91% (73/80)	99% (79/80)
Creek water	93% (28/30)	97% (29/30)	80% (16/20)	80% (16/20)
Sediment	60% (18/30)	57% (17/30)	43% (17/40)	30% (12/40)
Human stool	83% (25/30)	97% (29/30)	80% (24/30)	90% (27/30)
Fluorinated potable water	71% (40/56)	95% (53/56)	63% (35/56)	73% (41/56)
Extraction blank	83% (25/30)	97% (29/30)	80% (32/40)	83% (33/40)
**Target**				
*Bacteroides*	85% (23/27)	85% (23/27)	82% (22/27)	85% (23/27)
*Campylobacter jejuni, C coli*	77% (24/31)	87% (27/31)	49% (18/37)	78% (29/37)
*Cryptosporidium*	77% (24/31)	90% (28/31)	70% (26/37)	54% (20/37)
EPEC (*eae*)	74% (23/31)	90% (28/31)	73% (27/37)	78% (29/37)
*Giardia*	90% (28/31)	97% (30/31)	82% (22/27)	82% (22/27)
*Salmonella*	84% (26/31)	97% (30/31)	78% (25/32)	69% (22/32)
STEC (*stx1*)	85% (23/27)	96% (26/27)	88% (28/32)	91% (29/32)
STEC (*stx2*)	77% (24/31)	94% (29/31)	78% (29/37)	92% (34/37)

Data are % (n/N). Results are shown by sample matrix and by target. Sensitivity is defined as the percentage of spiked targets that were detected. Accuracy is measured as percentage of assays within one log_10_ of the spiked concentration; assays where background levels of pathogen were detected by qPCR or TAC are excluded from these calculations (20 creek water assays and ten human stool assays excluded). TAC=TaqMan array card. STEC=Shiga toxin-producing *Escherichia coli*. EPEC=enteropathogenic *Escherichia coli*.

The second set of test samples was used to establish the performance of each technique on samples with varying levels of PCR inhibition (appendix 2). For both methods, there was a reduction in sensitivity (for TAC, 77% [136/176] and for qPCR 89% [157/176]) and quantitation accuracy (for TAC 67% [124/186] and for qPCR 69% [129/186] of assays within one log of the known concentration) across all sample matrices (table 2). This decrease in performance compared with samples spiked in nuclease-free water (figure 1) suggests both methods, especially TAC, are affected by PCR inhibitors. There was nevertheless much variability in the relative performance of the two methods across different sample matrices and pathogen targets. For example, although TAC underperformed compared with qPCR in spiked fluorinated potable water samples, the converse was true for sediment samples. Likewise, although TAC detected *Cryptosporidium* with higher accuracy, qPCR was more sensitive and accurate for detecting *Campylobacter* (table 2). TAC also detected a range of indicators and pathogens present in Melbourne sewage and stormwater samples (detailed below). TAC was more inhibited by this sample matrix than qPCR and detected no targets (including universal 16S rRNA) in five of the eight samples. However, diluting samples (1:10 and 1:20) greatly improved detection for all samples, resulting in 216-fold and 273-fold increases in target quantities respectively (appendix 2).

**Figure 2:**
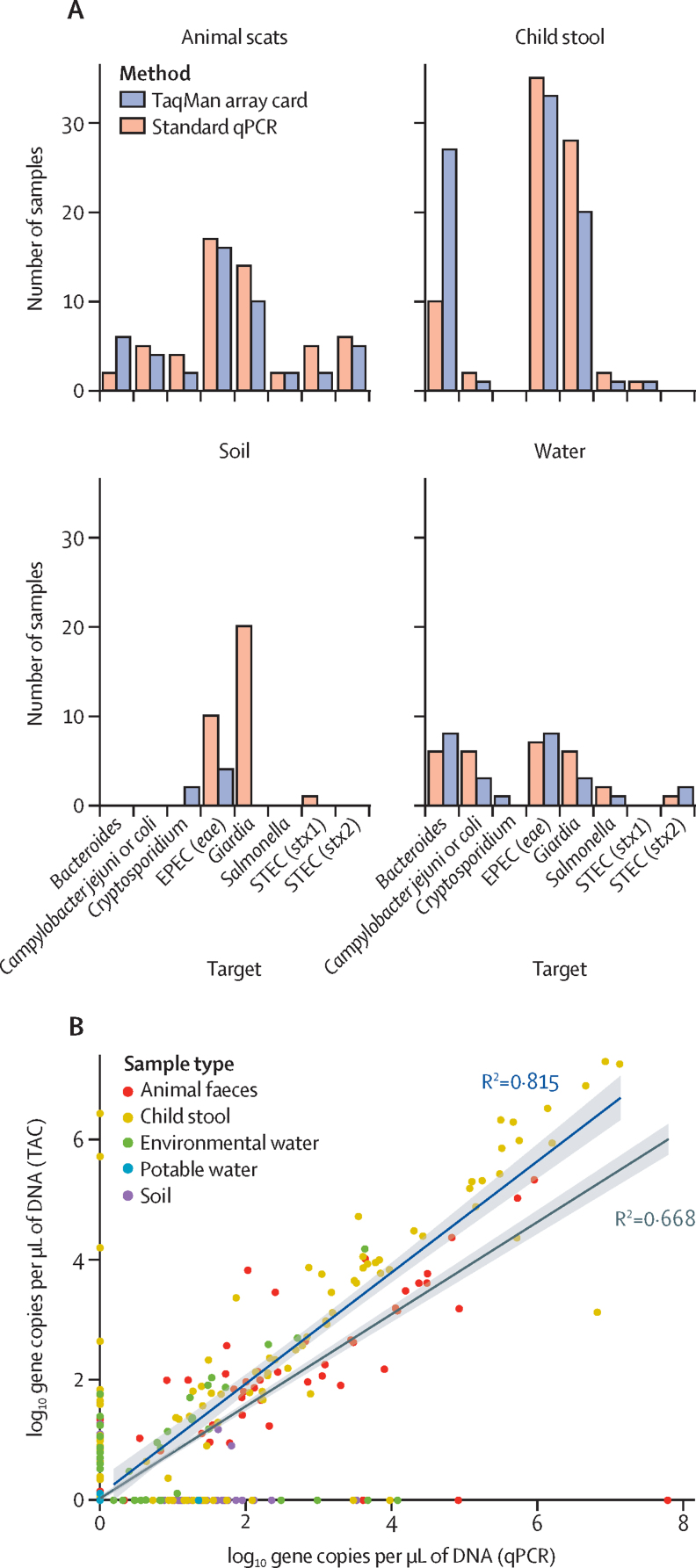
Concordance between standard qPCR and TAC in detecting pathogens in animal scats, child stool, soil, and water collected from informal settlements of Suva, Fiji Agreement between the methods with respect to the number of positive detections of targets (A) and the measured target quantity in log_10_ gene copies per μL of extracted DNA (with a pseudocount of 1 added before log_10_ transformation; (B). The regression lines with associated 95% CIs are shown for the subset of data where a target was quantified by both methods (blue, R^2^ 0·815). Across all datapoints, R^2^ 0·668 (grey). TAC=TaqMan array card. EPEC=enteropathogenic *Escherichia coli*. STEC=Shiga toxin-producing *Escherichia coli*.

A set of 121 samples from informal settlements in Fiji consisting of 60 child stool, 17 animal scats (predicted to be primarily from dogs and ducks), 20 water (ten environmental, ten potable), and 24 soil samples were analysed with TAC and standard qPCR. The nature and distribution of enteropathogen contamination in this environment are relatively unknown, and this sampling effort represents an initial insight into the baseline conditions of these settlements before the water and sanitation intervention to be trialled by the RISE programme.^34^, ^35^ The full dataset containing measured gene copies per μL is provided in appendix 2. For the eight pathogen targets assayed by both methods, the concordance rate between presence and absence was high, with 857 (89%) of 967 assays in agreement between both methods (Cohen's κ 0·619; figure 2A). Of the remainder, 70 (7%) represented a detection by qPCR that was not observed with TAC, and 40 (4%) represented a TAC detection missed by qPCR; this finding indicates that the greater overall sensitivity of qPCR does not preclude the ability for TAC to detect pathogens when qPCR does not. Only one sample (a child stool) was indicated to be substantially inhibited by the qPCR *Bacteroides* internal amplification control; despite this result, both methods detected the *Bacteroides* faecal indicator.

For assays where both methods detected the target, quantitation was quite consistent with an R^2^ of 0·815 (figure 2B). The distribution of measured quantities for targets detected by only one method is similar on both axes, indicating that both qPCR and TAC can similarly detect targets that are missed by the other method. The target quantities measured by the two methods differed significantly (p=0·00006), driven by instances where a target at low concentration was detected by one method and not the other (figure 2B). When considering the concordance between the techniques when a quantity was measured by both, differences in quantities were not statistically significant (p=0·21).

The pathogens detected in each sample type are shown in figure 2A. All targets were found in at least four samples. *Giardia* and enteropathogenic *Escherichia coli* (EPEC; *eae* gene) were the most common of the eight targets, whereas *Salmonella* and *Cryptosporidium* were found infrequently. Reflecting the concordance rate, detections of each target in each sample type were similar. The generic faecal indicator *Bacteroides* was detected more often by TAC than by qPCR, especially in child stool samples. The major discrepancy in results was the reported detection of *Giardia* in several soil samples by qPCR (quantified at 10–100 copies per μL of original sample), which were not detected by TAC. Negative extraction controls were free of amplification, except for a low concentration of *Giardia* in the animal scat control (detected by both methods) and in the soil control (detected only by qPCR). It is possible that these hits are true positives that were not detected by TAC due to a combination of low *Giardia* levels, sample dilution, and challenging detection in a soil matrix. However, it is also possible that false-positive detection underlies these issues given *Giardia* qPCRs accounted for the only false positive detected in the spiking study (figure 1).

**Figure 3:**
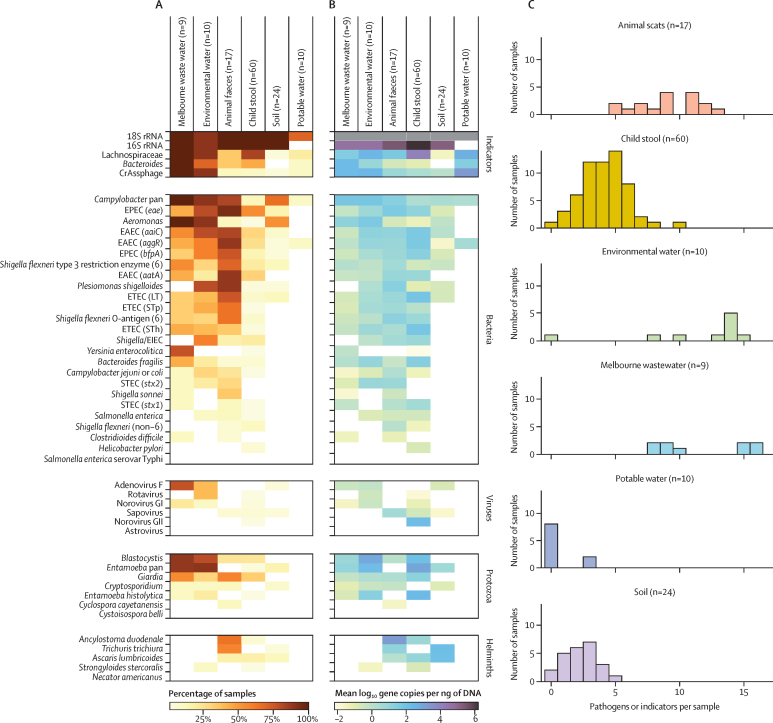
Pathogen and indicator targets detected via TAC in Melbourne wastewater samples and animal scats, child stool, soil, and water collected from informal settlements of Suva, Fiji Heatmaps represent the prevalence (percentage of positive samples [A]) and abundance (mean value of log_10_ gene copies per ng of DNA across positive samples [B]) of each target by sample type. White represents a zero value, and 18S rRNA quantitation was unavailable. The number of pathogens or indicators detected per sample is represented by histograms (C), also by sample type. This excludes the 16S rRNA and 18S rRNA targets and counts pathogens with multiple gene targets (ie, *Campylobacter* spp*, Shigella* spp, EAEC, ETEC, STEC, EPEC and *Entamoeba* spp) only once. TAC=TaqMan array card. EAEC=enteroaggregative *Escherichia coli*. ETEC=enterotoxigenic *Escherichia coli*. STEC=Shiga toxin-producing *Escherichia coli*. EPEC=enteropathogenic *Escherichia coli*.

In addition to the eight targets assayed via both methods, the custom TAC was designed to detect a range of other viral, bacterial, protist, and helminth enteropathogen targets (appendix 1 pp 5, 8). Of the 48 targets on the card, 44 were detected at least once (39 pathogen targets, three faecal indicators, two controls); astrovirus, *Salmonella enterica* serovar Typhi (typhoid fever), *Necator americanus* (hookworm), and *Cystoisospora belli* (isosporiasis) were not detected in any sample. Overall, most samples contained a wide range of enteropathogens (figure 3), except for potable water which contained only human faecal indicators at minimal concentrations. Environmental water and animal scat samples were richest in enteropathogens, with most samples containing more than eight pathogens and ten targets (figure 3C). Somewhat fewer pathogens were detected in child stool (mean 2·8 [SD 1·6] pathogens per sample) and soil (2·2 [SD 1·3]), excluding faecal indicators.

The most prevalent enteropathogens across host and environmental reservoirs were enteroaggregative *E coli* (EAEC) and EPEC. All three target genes were commonly detected for EAEC (*aaiC, aatA, aggR*), whereas the *eae* gene was detected more frequently than *bfpA* for EPEC (figure 2A). Both markers of *Shigella flexneri* clade 6 (O-antigen, type 3 restriction enzyme) were also present in 18 samples.^14^ *Giardia* and *Blastocystis* were common protists, with *Giardia* present at highest concentrations in human stool (figure 2B). Among helminths, the large roundworm *Ascaris* was most common in human faecal samples, which is concordant with findings that this genus infects approximately a sixth of the world's population.^46^ In contrast, *Ancylostoma* and *Trichuris* were predominant in animal faeces. Few helminths were detected in soil, but those that were present had moderately high abundance (approximately 120–140 copies per ng of DNA). Viruses were less prevalent overall; rotavirus and adenovirus F were most commonly detected, primarily in environmental water, whereas norovirus GII was abundant in one stool sample (appendix 2). Some other targets, notably *Campylobacter* spp, *Entamoeba* spp, *Aeromonas*, and *Plesiomonas shigelloides*, were abundant in environmental waters and other samples. However, they were found infrequently in child stool.

TAC also detected a range of faecal indicators and other marker genes. The universal bacterial marker 16S rRNA was detected in most samples, and was absent in only one environmental water and all potable water samples. Providing an estimate of total bacterial load, 16S rRNA quantities were highest in the human faecal samples and lowest in environmental water. Aside from the universal 16S rRNA assay, the most common target detected overall was human-associated Lachnospiraceae, a faecal marker detected in the majority of child stool and environmental water samples. CrAssphage, an abundant bacteriophage of human *Bacteroides* and a proposed faecal indicator,^41^, ^47^ was the least common faecal indicator detected in individual stool samples, but was present in most of the environmental water samples. These reportedly human-specific faecal indicators were also detected in the animal scats, though to a lesser extent than in child stool (figure 2).

## Discussion

The TAC has been widely used for pathogen detection in human clinical samples,^26^, ^30^, ^31^ but has, to the best of our knowledge, been used in only two studies to date to detect enteropathogens in non-human samples.^18^, ^33^ In the current study, we compared enteropathogen detection via TAC and standard qPCR using spiked samples of known concentration, wastewater samples from Melbourne, Australia, and a range of sample types collected from urban informal settlements in Fiji. Overall, we found that the performance of TAC in environmental samples was reduced but comparable to standard qPCR. The capacity of TAC to efficiently detect multiple enteropathogen targets potentially counterbalances the trade-offs in sensitivity and accuracy, given the benefits of monitoring a large array of enteropathogens in samples from heavily contaminated environments. In addition, we show that TAC can effectively quantify enteric pathogens across a range of environmental, human, and animal reservoirs, thereby providing a unified method to monitor pathogen transmission pathways and evaluate public health interventions.

It can be expected that TAC is less sensitive than qPCR. The smaller reaction volume for TAC (approximately 1 μL compared with the 20 μL standard for qPCRs) corresponds to a reduced chance that a reaction well contains a copy of a low-concentration target. Reduction in sensitivity has been observed in previous comparisons between standard qPCR and TAC, the extent of which can vary by assay.^30^, ^31^, ^48^ Importantly, some of these previous comparisons have evaluated the performance of TAC relative to standard qPCR, rather than a side-by-side comparison.^31^, ^48^ In this context, the sensitivity and accuracy of standard qPCR is assumed to be 100% (as the gold standard) and TAC performance is reported as the percentage of assays that agree with standard qPCR results. Our tests with spiked environmental sample matrices indicated that the performance of standard qPCR is not optimal; it generally overestimated target abundance and had reduced sensitivity in some matrices (eg, fluorinated water, sediment). By evaluating both methods independently, we provide a clearer view of how they each perform in challenging sample types and demonstrate that, although TAC is overall less sensitive and accurate than standard qPCR, it is highly comparable for use in these conditions. Array card sensitivity might be further improved by designing cards with fewer targets spotted in duplicate: Liu and colleagues^26^ reported that almost half of low-concentration targets spiked into stool were detected in only one of two replicates, similar to the variability observed at the lowest dilution of our TAC standard curve.

TAC was generally more susceptible to inhibition than standard qPCR in the mock and Melbourne wastewater samples. However, 1:10 sample dilution greatly reduced inhibition without compromising detection for moderately to highly abundant targets, in common with previous findings.^18^, ^33^ By contrast, we detected minimal inhibition in the Fijian stool, scat, soil, and water samples, as verified by the standard qPCR internal control. We recommend that environmental studies test for PCR inhibition before application of TAC to establish how best to reduce this (eg, dilution or re-extraction of samples with optimised methods). Both extraction efficiency and qPCR reaction inhibition could be monitored on TAC by adding amplification controls before extraction (eg, MS2 and PhHV viruses).^26^ However, we elected not to take this approach due to concerns that the addition of control DNA or RNA would impact the detection of low concentration targets in already low-biomass environmental samples. Instead, we included a universal 16S rRNA target in addition to the manufacturer's 18S rRNA control; failure of these targets to amplify indicates either substantial inhibition or minimal sample biomass, which can be distinguished by DNA quantification.

Using the same fundamental method of TaqMan qPCR, both standard qPCR and TAC are excellent options for environmental enteropathogen monitoring. As summarised in table 3, they have different strengths and limitations depending on purpose of their applications. Overall, standard qPCR offers greater sensitivity, flexibility, and the ease of running replicates to improve confidence in positive results and quantities. TAC can detect more than 45 custom targets across eight samples in a minimally laborious way, eliminating pipetting error, and greatly reducing the potential for assay contamination. An overarching caveat is that TAC is designed to provide the best overall result and will not be optimal for individual pathogens. Additionally, as TAC standard curves require one whole card per replicate, quantitation is less stringent. We demonstrated with spiked gene blocks that accurate quantitation with TAC is achievable, but quantitation is complicated by detection of DNA and mRNA with the universal reverse transcription step and assays that target multicopy genes. However, in the context of water and sanitation interventions, a consistent method measuring relative change over time without the need to calculate organism numbers or identify aetiology is appropriate, and the approach potentially boosts sensitivity. Although the analysis of samples via TAC is cost-effective per-sample-per-pathogen, there is a high upfront cost to purchasing the QuantStudio 7 or ViiA 7 array equipment, which could be prohibitive. Additionally, if the panel requires redesign (eg, the addition of new pathogen targets), a new batch of array cards must be purchased; a benefit of standard qPCR is that assays are run individually and targets can be changed at any time. A limitation inherent to both methods is the need to dilute samples to reduce PCR inhibition. This approach runs the risk of diluting targets beyond the limit of detection, although it might be resolved to some extent by optimised DNA extraction and purification methods.

**Table 3:** Comparison of TAC and qPCR for monitoring multiple pathogens

	**qPCR and RT-qPCR**	**TAC**
Target range	Narrow; individually detects any target of interest with appropriately optimised primers or probes; however, adding additional targets requires extra sample volume, labour, cost, and plastic waste; assays can be multiplexed (detection of multiple targets in one reaction) with careful optimisation	Broad; simultaneously detects up to 47 singleplex targets and 1 internal control across 8 samples; wells can be multiplexed or samples can be reduced to increase target number; assays require careful card design and manufacture with appropriate lead-time; optimisation may be required for target quantitation under universal conditions on the card
Sensitivity, accuracy	High sensitivity and accuracy; theoretical detection limit is approximately three gene copies per reaction; pathogen quantitation possible with appropriate reference standards; PCR inhibition possible, but can be readily monitored with controls	High sensitivity, medium accuracy; sensitivity high but often lower than qPCR given smaller reaction volume and universal reaction conditions; pathogen quantitation possible with reference standards, but generally requires comparisons between cards and typically a positive control plasmid containing multiple primer and probe sequences in tandem; quantitation also challenging due to co-detection of DNA and RNA due to universal reverse transcriptase step to detect RNA viruses; greater potential for PCR inhibition
Specificity	High; well designed TaqMan primer and probe sequences are very specific	High; same TaqMan technology as standard qPCR
Scalability	Moderate; extensive manual handling with large numbers of samples or pathogens; large sample numbers require high labour time or robotics; increased sample numbers require greater sample volume and produce more waste; high potential for pipetting errors	High; simple and moderately fast (approximately 3 h) to prepare and run from extracted nucleic acids; labour time is minimal given the few manual handling tasks required, though increases per card (eight samples); low potential for pipetting errors
Flexibility	High; given assays are run individually, targets can be changed at any time; reaction conditions can be modified to optimise amplification of each target	Low; a new set of cards must be manufactured and validated to add or change targets; the same reaction conditions must be used for each target
Cost	Low cost per sample; low reagent cost per sample (approximately US$2·10 for one pathogen without replicates); small cost increase with more samples, but large increase with more targets (double the labour and reagents cost for two targets); high upfront cost of real-time thermal cycler	Low cost per pathogen; moderate reagent cost per sample (approximately US$60); however, highly cost-effective for monitoring multiple targets per sample (approximately US$1·28 per sample per target without replicates); high upfront cost of real-time thermal cycler with array card block
Resources	Moderate; requires real-time thermal cycler; training for molecular biology, equipment use, and software required	Moderate; requires real-time thermal cycler with array card block; training needed for molecular biology, equipment, and software

RT-qPCR=reverse transcriptase qPCR. TAC=TaqMan array cards.

We observed a diverse range of enteropathogens in urban informal settlements in Suva, Fiji, highlighting the utility of TAC in this setting. There was a high burden of bacterial pathogens in human stools, particularly EAEC and EPEC, as well as a wide range of bacterial, protist, and helminth pathogens in environmental waters and animal scats. This is a similar finding to that of Baker and colleagues,^18^ who also observed widespread diarrhoeagenic *E coli* in soil and water from neighbourhoods in Kisumu, Kenya, although they also detected a much higher prevalence of *Cryptosporidium.* They also reported similar numbers of pathogens per sample, with particularly high pathogen diversity observed in environmental waters,^18^ as observed in Fiji. The inclusion and detection of soil-transmitted helminths in both human and environmental samples on TAC is a particularly important advance, as the standard methods for detection of such pathogens in stool involve conventional microscopy (labour-intensive, subjective, and high risk for the operator) or serology (only viable for human-derived samples).^49^ Despite their transmission pathway, soil-transmitted helminths were more common in human and animal faeces than in soil samples from Fiji. Our soil samples might not have been representative of helminth-contaminated areas; detection might have also been influenced by the integrity of helminth eggs impeding DNA extraction, difficulties of the soil matrix (demonstrated by our mock samples) and the small volume of soil extracted. In agreement with previous studies,^39^, ^50^ Lachnospiraceae appears to be a useful faecal indicator in this population as it was detected in the majority of faecal samples. As the assay is reportedly human-specific,^39^ its presence in animal scats suggests a close association between animals and people in these settings. Although *Bacteroides* and CrAssphage were less frequently detected in individual child stools, all three indicators were common in environmental waters. Through RISE, we are doing further studies to understand the distribution and transmission of enteropathogens in the Fiji sites, as well as the trial settlements in Makassar, Indonesia.^35^

This study nevertheless has several limitations. First, we did side-by-side comparisons of only eight of the 48 targets on the TAC, reflecting that it is logistically prohibitive to quantify numerous targets across multiple samples with standard qPCR. Second, it should be noted that the number of PCR cycles was not the same between the two methods (40 cycles for qPCR *vs* 45 cycles for TAC) because we followed an optimised protocol for each method. Although this approach imposes a limit on what can be detected by qPCR and extends the opportunity for false positives via TAC for five cycles, TAC C_q_ values above 40 were rare across the study (28/1712). When using TAC in wider studies, we recommend a cautious approach to the interpretation of results at higher cycle numbers; for example, values between C_q_ 40 and 45 could be considered false positives, and values between the assay LLOQ and C_q_ 40 could be interpreted as “inconclusive”. Finally, an important consideration when interpreting qPCR-based pathogen detection is that it does not indicate organism viability. Persistence of DNA from non-viable pathogens in the environment is likely to vary by organism, and presence of DNA in stool samples without active clinical infection can also complicate interpretation of results.^51^

To conclude, techniques that can adequately monitor a range of enteropathogens in humans, animals, and the environment are required to assess water and sanitation improvements that aim to interrupt diverse transmission pathways. The use of qPCR in the form of individual assays or via the TAC enables direct detection of several enteropathogens in a range of sample types, bypassing reliance on faecal indicator organisms. Our study is the first to our knowledge to evaluate the performance of a custom TAC compared with standard qPCRs on human, animal, and environmental samples. We have demonstrated that, in these challenging sample matrices, TAC is comparable to standard qPCR and is a cost-effective, scalable, accurate, and easy to use alternative for multiple pathogens. Better understanding of the distribution, transmission, and impacts of a broad range of enteropathogens across environmental, human, and animal reservoirs is essential for improvements to public health towards SDGs 3 and 6. Among various potential applications, this research will be crucial for informing and evaluating future water and sanitation interventions in urban informal settlements, where the nature and extent of enteropathogen contamination is poorly characterised and diverse. More broadly, this technology enables unified approaches for surveying enteropathogens in populations and environments, as well as resolving and interrupting their transmission pathways.

## Data sharing

The data analysed in this study (all results expressed in gene copies per μL of undiluted genomic DNA) are available in appendices 1 and 2. The protocol for the wider RISE study is available online. Additional details on the qPCR and TAC data or analyses are available from the authors upon request.

## Declaration of interests

We declare no competing interests.
