# Correction *Lancet Planet Health* 2021; 5: e297–308

**DOI:** 10.1016/S2542-5196(21)00139-X

**Published:** 2021-05-19

*Lappan R, Henry R, Chown SL, et al. Monitoring of diverse enteric pathogens across environmental and host reservoirs with TaqMan array cards and standard qPCR: a methodological comparison study.* Lancet Planet Health *2021;* 5: *e297–308*—In this Article, the Summary Methods section should have stated that “Both methods exhibited similarly *high* specificity”. This correction has been made as of May 18, 2021.

