# Supplementary material for: Monitoring of diverse enteric pathogens across environmental and host reservoirs with TaqMan array cards and standard qPCR: a methodological comparison study
Source: Lancet Planet Health. 2021 May 5;5(5):e297–308. doi: 10.1016/S2542-5196(21)00051-6 (PMC8116308; doi:10.1016/S2542-5196(21)00051-6)
Supplement: Supplementary appendix 1 [file mmc1.pdf]

THE LANCET
Planetary Health

Supplementary appendix 1

This appendix formed part of the original submission and has been peer reviewed. We post it as supplied by the authors.

Supplement to: Lappan R, Henry R, Chown SL, et al. Monitoring of diverse enteric pathogens across environmental and host reservoirs with TaqMan array cards and standard qPCR: a methodological comparison study. *Lancet Planet Health* 2021; **5:** e297–308.

Supplementary Appendix for:

Monitoring of diverse enteric pathogens across environmental and host reservoirs with TaqMan array cards and standard quantitative PCR: a methodological comparison study

Rachael Lappan PhD[1,2*], Rebekah Henry PhD[3*], Prof. Steven L. Chown PhD[2], Prof. Stephen P. Luby MD[4], Ellen E. Higginson PhD[5], Lamiya Bata BSc (Hons)[3], Thanavit Jirapanjawat BSc (Hons)[1,2], Christelle Schang MEng[3], John J. Openshaw MD[4], Joanne O'Toole PhD[6], Audrie Lin PhD[7], Autiko Tela BSc[8], Amelia Turagabeci PhD[8], Prof. Tony H.F. Wong PhD[9], Matthew A. French PhD[10], Prof. Rebekah R. Brown PhD[10], Prof. Karin Leder PhD[6], Chris Greening PhD[1,2#], David McCarthy PhD[3#]

1. Department of Microbiology, Biomedicine Discovery Institute, Monash University, Clayton, VIC 3800, Australia
2. School of Biological Sciences, Monash University, Clayton, VIC 3800, Australia
3. Department of Civil Engineering, Monash University, Clayton, VIC 3800, Australia
4. Division of Infectious Diseases and Geographic Medicine, Stanford University, Stanford, CA 94305, USA
5. Cambridge Institute for Therapeutic Immunology and Infectious Disease, University of Cambridge, Cambridge CB2 0AW, United Kingdom
6. School of Public Health and Preventive Medicine, Monash University, Melbourne, VIC 3004, Australia
7. Division of Epidemiology and Biostatistics, School of Public Health, University of California Berkeley, CA 94720, USA
8. School of Public Health, Fiji National University, Suva, Fiji
9. Water Sensitive Cities Institute, Monash University, Clayton, VIC 3800, Australia
10. Monash Sustainable Development Institute, Monash University, Clayton, VIC 3800, Australia

Supplementary methods

Spiked sample preparation

Dilution of gene blocks for addition to each test matrix was conducted based on conversion of the measured nanograms of resuspended amplicon to total gene copy number using the formula:

$$\text{number of copies (molecules)} = \frac{X \text{ ng} * 6.0221 \text{ x } 10^{23} \text{ molecules/mole}}{(N * 660\text{g/mole}) * 1 \text{ x } 10^{9} \text{ ng/g}} \quad (1)$$

where X is the amount of measured amplicon (ng), N is the total length of dsDNA amplicon and 660 g/mole represents the average mass of 1 bp dsDNA.

Set 1 of mock samples (ten samples) consisted of the following: three samples contained all eight targets at low (10 copies/µl), medium (100 copies/µl), or high (1000 copies/µl) concentration; six samples contained combinations of targets and concentrations; and one sample was a blank with no targets spiked. See **Table S2** for details. Set 2 of mock samples (36 samples) included: nine wastewater samples with no gene blocks spiked; seven potable water samples spiked with 200 copies/µl of each target; and five different combinations of low, medium and high spiked targets in extracted DNA from each of four additional matrices (creek water, human stool, sediment and DNA extraction blank), including a background test with no gene blocks spiked. These samples were extracted in the same manner as the Fiji samples, below.

As initial testing indicated that TAC reactions were inhibited for the wastewater and potable water matrices, these samples were assayed with TAC undiluted, diluted 1:10, and diluted 1:20 with nuclease-free water. Where these results are reported, the C_q values from the 1:10 dilution are used to calculate the gene copies per microlitre in the undiluted DNA sample as this level of dilution was most effective at resolving inhibition.

Fiji sample collection and processing

Sixty child (< 5 year-old) stool samples were randomly selected for the current study from a total of 287 samples collected from 12 informal settlements during the period September 27 to November 8 2019. Samples were collected by the caregiver and stored at 4°C on frozen gel packs prior to transport to the laboratory and storage at -80°C within 24-48 hours.

Twenty water samples were collected in clean, source-water rinsed disposable bottles from the associated settlement. Potable water was run from local municipal water sources for 1 min prior to direct collection of 2 L of sample. Riverine, freshwater and stormwater (environmental water) samples were taken perpendicular from the bank and at an approximate depth of 0.15 m at each location. For each potable and environmental water sample, 1 L was filtered where possible through five 0.22 µM filters (Millipore). Where sediment prevented the passing of 1 L, a reduced volume was filtered until a total of five filters were collected. Filters were stored at -80°C within food-grade sealable bags.

Soil samples were collected using a sterile tongue depressor to transfer 2 cm^3 of material into food-grade sealable bags. Total animal stools were collected and stored in the same manner, with visual assessment of stool age to prevent collection of older "dry" samples. Material was placed at 4°C and transferred to the laboratory within two to four hours of collection. Animal scats and soil samples were homogenised (Stomacher 400 circulator, Seward) for 1 min at 250 × rpm and stored in 0.25 g aliquots in sterile cryo-storage tubes at -80°C.

For child stool, animal scats and soil samples, total genomic DNA was isolated from 0.25 g of material using the QIAGEN DNeasy PowerSoil Pro kit as per manufacturer's instructions, and eluted in 50 µL of

sterile molecular grade water. For water samples, the filters were crushed within the bags and transferred to the bead tubes with disposable spatulas for extraction with the QIAGEN DNeasy PowerMax Soil kit with the following modifications: after the addition of buffer C1, the samples were incubated at 65°C with shaking for 30 min at 200 rpm to lyse bacterial cells. The membranes were incubated for 10 min at room temperature in 1.5 ml of nuclease-free water prior to elution in this volume. Viral RNA is co-extracted with these kits as there is no RNase step.

Standard TaqMan qPCR detection

Each 25 µL reaction contained 2 µL of either diluted standard gene block or sample genomic DNA. Reactions were conducted in triplicate for each sample and standard. Gene blocks were serially diluted 10-fold to achieve a five-point standard curve ranging from 10^5 to 10 copies/µL. Similarly, an internal amplification control gene block was diluted to a final concentration of 50 copies/µL with 100 copies added to each 25 µL reaction to indicate PCR inhibition. Six replicates of no template controls were included on each run. The *Sketa22* assay described in Method 1696 was not performed as salmon testes DNA was not added prior to sample extraction. Quality control, data analysis and calculations were conducted as outlined in Method 1696[36] (using https://www.epa.gov/sites/production/files/2019-04/methods-1696-1697-analysis-tool_march-2019.xltm), to ensure acceptance thresholds were met for R^2 (standards), amplification efficiency (*E*), no-template control (NTC), method blank, internal amplification control, and lower limit of quantification (*LLOQ*). Relative fluorescence units (RFU) analysis was conducted to ensure a peak had been generated for each target assay. The LLOQ details can be found in **Table S4**, where the LLOQ is defined as the lowest dilution of the standard curve at which 95% of all standard curve replicates across the study were positive.

TaqMan Array card detection

TaqMan Array Cards were loaded with 100 µl of reaction mix per port, containing 60 µl of AgPath-ID One-Step RT-PCR master mix (Applied Biosystems; 50 µl buffer, 4 µl enzyme mix and 6 µl nuclease-free water per port) mixed with 40 µl of sample nucleic acid.[26] Samples were diluted in nuclease-free water as necessary to allow a maximum of 1400 ng total nucleic acid per port, and 8 samples were tested per card. Loaded cards were centrifuged and sealed as per manufacturer's instructions, and run on a QuantStudio 7 Flex instrument (Applied Biosystems) under the following cycling conditions: 45°C for 20 minutes, then 95°C for 10 minutes, followed by 45 cycles of 95°C for 15 seconds and 60°C for 1 minute.[26]

For positive control material, three plasmids were designed containing all TAC primer and probe sequences in inserts of approximately 1 kb each (15-20 targets per plasmid). If the primers or probe were degenerate, the sequence from the reference genome was used. The three plasmid controls were combined at equal concentrations and seven 10-fold serial dilutions were used to make the standard curve (7.2×10^6 to 7.2 copies per microlitre prior to adding to the master mix, corresponding to 2.88×10^6 to 2.88 copies per microlitre on the card). This set of dilutions (seven points on a standard curve plus a no-template control) were run in triplicate, across three cards. Within the QuantStudio analysis software, the threshold line for determining cycle threshold (C_q) was manually adjusted per target where necessary (when the software's automatic threshold was placed inappropriately for the amplification curves produced), with C_q values exported to calculate a linear equation per target from the three replicates in R v3.6.2.[43] This standard curve was used to quantify gene copies across the study. Note that a standard curve could not be generated for the manufacturer's 18S rRNA control.

For quality control, a sample containing one of the positive control plasmids (not containing the rotavirus assay) mixed with rotavirus A genomic RNA was run when each new batch of master mix reagents were first used, to test DNA polymerase and reverse transcriptase activity. Additionally, a no-template control (containing the same nuclease-free water as used to dilute samples on that card) was included once every 10 cards to monitor reagent contamination.

Within the analysis software, samples flagged with BADROX and NOISE or SPIKE, or another flag precluding the calculation of an appropriate C_q value were omitted from analysis. The threshold line was then adjusted per target, if necessary, to achieve an appropriate threshold for all positive amplification curves for that target. This was only done if the automatically determined threshold was inappropriately placed.

Figure S1. Layout of the custom TaqMan Array Card (TAC) designed and optimised for this study. Each card contains eight ports. Each port is connected to 48 wells each containing a different set of primer pairs and probes specific to the listed pathogen or indicator.

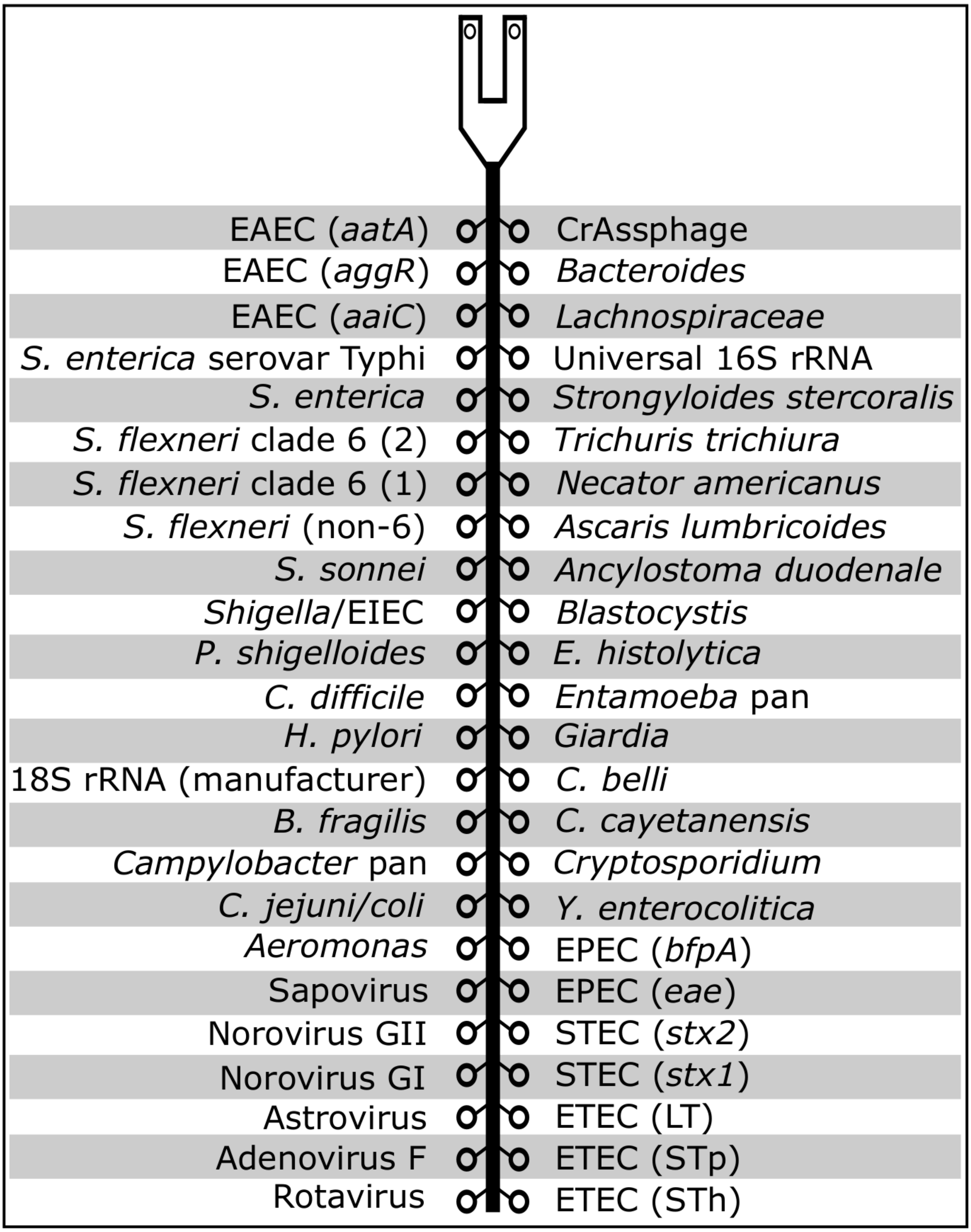

Table S1. Gene block fragments applied to the spiked test samples in this study.

Organism	Target	Length (bp)	Sequence
Campylobacter jejuni	*cadF*	221	CTTTGAAGGTAATTTAGATATGGATAATCGTTATGCACCAGGGATTAGACTTGGTTATCATTTTGACGATTTTTGGCTTGATCA ATTAGAATTTGGGTTAGAGCATTATTCTGATGTTAAATATACAAATACTAATAAAACTACAGATATTACAAGAACTTATTTGAG TGCTATTAAAGGTATTGATGTAGGTGAGAAATTTTATTTCTATGGTTTAGCAG
Salmonella enterica	*invA*	140	GCCGATGCCGGTGAAATTATCGCCACGTTCGGGCAATTCGTTATTGGCGATAGCCTGGCGGTGGGTTTTGTTGTCTTCTCTATT GTCACCGTGGTCCAGTTTATCGTTATTACCAAAGGTTCAGAACGTGTCGCGGAAGT
Escherichia coli	*eae*	180	CCCGCTTTACGGCAAATTTAGGTGCGGGTCAGCGTTTTTTCCTTCCTGAAAATATGTTGGGCTATAACGTCTTCATTGATCAGGA TTTTTCTGGTGATAATACCCGTTTAGGTATTGGTGGCGAATACTGGCGAGACTATTTCAAAAGTAGTGTTAACGGCTATTTCCGC ATGAGCGGCT
Escherichia coli	*stx-1*	132	ACTTCTCGACTGCAAAGACGTATGTAGATTCGCTGAATGTCATTCGCTCTGCAATAGGTACTCCATTACAGACTATTTCATCAGGA GGTACGTCTTTACTGATGATTGATAGTGGCACAGGGGATAATTTGT
Escherichia coli	*stx-2*	180	CTCTTCGTTAAATAGTATACGGACAGAGATATCGACCCCTCTTGAACATATATCTCAGGGGACCACATCGGTGTCTGTTATTAACC ACACCCCACCGGGCAGTTATTTTGCTGTGGATATACGAGGGCTTGATGTCTATCAGGCGCGTTTTGACCATCTTCGTCTGATTATT GAGCAAAA
Cryptosporidium parvum	*18S rRNA*	125	GGGTTGTATTTATTAGATAAAGAACCAATTTATTGGTGACTCATAATAACTTTACGGATCACATTAAATGTGACATATCATTCAAGT TTCTGACCTATCAGCTTTAGACGGTAGGGTATTGGCCT
Giardia lamblia	*18S rRNA*	89	TCACCCGGGACGCGGCGGACGGCTCAGGACAACGGTTGCACCCCCCGCGGCGGTCCCTGCTAGCCGGACACCGCTGGCAACCCG GCGCC
Bacteroides spp.*	*16S rRNA*	127	ATCATGAGTTCACATGTCCGCATGATTAAAGGTATTTTCCGGTAGACGATGTGTAGCAACGGCGTGTTATAGTAGGCGGGGTAAC GGCCCACCTAGTCAACGATGGATAGGGGTTCTGAGAGGAAGG
Bacteroides spp	*16S rRNA*	133	ATCATGAGTTCACATGTCCGCATGATTAAAGGTATTTTCCGGTAGACGATGGGGATGCGTTCCATTAGCTCGAGATAGTAGGCGG GGTAACGGCCCACCTAGTCAACGATGGATAGGGGTTCTGAGAGGAAGG

**Bacteroides* spp. internal amplification control

Table S2 (xlsx) – see Appendix 2. Details of the samples spiked for the comparison of performance of TAC and standard qPCR. The first tab provides details of the targets spiked in nuclease-free water. The second tab provides details of the targets spiked in different matrices containing potential PCR inhibitors.

Table S3. List of primers and probes present in the TaqMan array card. All selected primers and probes were based on the cited references.

Organism	Target gene	Forward primer	Reverse primer	Probe (all 5'FAM 3'MGB)
Rotavirus[1,2]	NSP3	ACCATCTWCACRTRACCCTCTATGAG	GGTCACATAACGCCCCTATAGC	AGTTAAAAGCTAACACTGTCAAA
Adenovirus 40/41 (F)[2]	Fiber	AACTTTCTCTCTTAATAGACGCC	AGGGGGCTAGAAAACAAAA	CTGACACGGGCACTCT
Astrovirus[1,2]	Capsid	CAGTTGCTTGCTGCGTTCA	CTTGCTAGCCATCACACTTCT	CACAGAAGAGCAACTCCATCGC
Norovirus GI[2]	ORF1-2	CGYTGGATGCGNTTYCATGA	CTTAGACGCCATCATCATTYAC	TGGACAGGAGATCGC
Norovirus GII[1,2]	ORF1-2	CARGARBCNATGTTYAGRTGGATGAG	TCGACGCCATCTTCATTCACA	TGGGAGGGCGATCGCAATCT
Sapovirus[1]	RdRp	GAYCAGGCTCTCGCYACCTAC<br>TTTGAACAAGCTGTGGCATGCTAC	CCCTCCATYTCAAACACTA	CYTGGTTCATAGGTGGTRCAG<br>CAGCTGGTACATTGGTGGCAC
Aeromonas[1]	Aerolysin	TYCGYTACCAGTGGGACAAG	CCRGCAAACTGGCTCTCG	CAGTTCCAGTCCCACCACTT
Campylobacter jejuni / coli[1,2]	*cadF*	CTGCTAAACCATAGAAATAAAATTTCTCAC	CTTTGAAGGTAATTTAGATATGGATAATCG	CATTTTGACGATTTTTGGCTTGA
Campylobacter pan[2]	*cpn60*	AAAGTIGGMAAAGATGGTGTTAT<br>AAAGTIGGWAAAGACGGYGTTAT	TCAAATTGCATACCYTCAAC	TTTGCCTCTTCMACAGT<br>TTTGCTTCTTCWACAGT
Bacteroides fragilis[2]	*bft*	GGGACAAGGATTCTACCAGCTTTATA	ATTCGGCAATCTCATTCATCATT	CAATGGCGAATCCATCAG
Helicobacter pylori[2]	*ureC*	GACACCAGAAAAAGCGGCTA	AGCGCATGTCTTCGGTTAAA	TCACTAAAGCGTTTTCTACC
Clostridioides difficile[1,2]	*tcdB*	GGTATTACCTAATGCTCCAAATAG	TTTGTGCCATCATTTTCTAAGC	CCTGGTGTCCATCCTGTTTC
Plesiomonas shigelloides[2]	*gyrB*	CCGCCGTGAAGGCAAAG	GCTACCGGCTCACCCAGAT	CACACCCAAGAATAC
Shigella/EIEC[1,2]	*ipaH*	CCTTTTCCGCGTTCCTTGA	CGGAATCCGGAGGTATTGC	CGCCTTTCCGATACCGTCTCTGCA
Shigella sonnei[3]	Putative methylase	TGCCGCTAAAATCCTTCTGT	GCGTACGACGAAAGGAAAAA	GAAGTTATTGATTCCGCCC
Shigella flexneri (most serotypes except 6)[3]	Putative periplasmic protein	TGGGTGCATCCTGACCTGT	GACAAACAATAACGAGCTACCGAT	ACCACGGAATAATCCCGCAG
**Shigella flexneri* 6[3]	O-antigen	CTCCTATCCGTGATTATAGTGCA	GCACACACAACTCACTGTATTT	TCCTTCTCACGATTAAAATC
**Shigella flexneri* 6[3]	Type 3 restriction enzyme	CTTTCAACGCACGAATATCAAC	GAACCTGATCCAGACGGAGA	TTCTTCAGAACCGGGTTTTG
Salmonella enterica[1]	*invA*	TCGGGCAATTCGTTATTGG	GATAAACTGGACCACGGTGACA	AAGACAACAAAACCCACCGC
Salmonella enterica serovar Typhi[2]	*staG* (STY0201)	CGCGAAGTCAGAGTCGACATAG	AAGACCTCAACGCCGATCAC	CAGCCTGCTCCAGAACA
EAEC[1,2]	*aaiC*	ATTGTCCTCAGGCATTTCAC	ACGACACCCCTGATAAACAA	TAGTGCATACTCATCATTTAAG
EAEC[2]	*aggR*	GCAATCAGATTAARCAGCGATACA	TTCGGACAACTRCAAGCATC	AAGACGCCTAAAGGATGCCC

EAEC[1,2]	*aatA*	CTGGCGAAAGACTGTATCAT	TTTTGCTTCATAAGCCGATAGA	TGGTTCTCATCTATTACAGACAGC
ETEC[1,2]	STh	GCTAAACCAGYAGRGTCTTCAAAA	CCCGGTACARGCAGGATTACAACA	TGGTCCTGAAAGCATGAA
ETEC[1,2]	STp	TGAATCACTTGACTCTTCAAAA	GGCAGGATTACAACAAAGTT	TGAACAACACATTTTACTGCT
ETEC[1,2]	LT	TTCCCACCGGATCACCAA	CAACCTTGTGGTGCATGATGA	CTTGGAGAGAAGAACCCT
STEC[1,2]	*stx1*	ACTTCTCGACTGCAAAGACGTATG	ACAAATTATCCCCTGWGCCACTATC	CTCTGCAATAGGTACTCCA
STEC[1,2]	*stx2*	CCACATCGGTGTCTGTTATTAACC	GGTCAAAACGCGCCTGATAG	TTGCTGTGGATATACGAGG
EPEC[1,2]	*eae*	CATTGATCAGGATTTTTCTGGTGATA	CTCATGCGGAAATAGCCGTTA	ATACTGGCGAGACTATTTCAA
EPEC[1,2]	*bfpA*	TGGTGCTTGCGCTTGCT	CGTTGCGCTCATTACTTCTG	CAGTCTGCGTCTGATTCCAA
Yersinia enterocolitica[2]	*lysP*	TGATTCACCAGCAGCAATAC	GGCATCATGAAAGGCGG	TGTCGGTTTCTCCTTCCAGG
Cryptosporidium[1,2]	18S rRNA	GGGTTGTATTTATTAGATAAAGAACCA	AGGCCAATACCCTACCGTCT	TGACATATCATTCAAGTTTCTGAC
Cyclospora cayetanensis[2]	18S rRNA	AAAAGCTCGTAGTTGGATTTCTG	AACACCAACGCACGCAGC	AAGGCCGGATGACCACGA
Cystoisospora belli[2]	ITS2	ATATTCCCTGCAGCATGTCTGTTT	CCACACGCGTATTCCAGAGA	CAAGTTCTGCTCACGCGCTTCTGG
Giardia[1,2]	18S rRNA	GACGGCTCAGGACAACGGTT	TTGCCAGCGGTGTCCG	CCCGCGGCGGTCCCTGCTAG
Entamoeba pan[2]	18S rRNA	AAACGATGTCAACCAAGGATTG	TCCCCCTGAAGTCCATAAACTC	CCTTGTTCAGAACTTAAAGAGAAA
Entamoeba histolytica[1,2]	18S rRNA	ATTGTCGTGGCATCCTAACTCA	GCGGACGGCTCATTATAACA	TCATTGAATGAATTGGCCATTT
Blastocystis[2]	18S rRNA	TGGTCCGRTGAACACTTTGGAT	CCTACGGAAACCTTGTTACGACTTCA	CTTCCTCTAAATGRTAAGATT
Ancylostoma duodenale[2]	ITS2	GAATGACAGCAAACTCGTTGTTG	ATACTAGCCACTGCCGAAACGT	ATCGTTTACCGACTTTAG
Ascaris lumbricoides[2]	ITS1	GCCACATAGTAAATTGCACACAAAT	GCCTTTCTAACAAGCCCAACAT	TTGGCGGACAATTGCATGCGAT
Necator americanus[2]	ITS2	CTGTTTGTCGAACGGTACTTGC	ATAACAGCGTGCACATGTTGC	CTGTACTACGCATTGTATAC
Trichuris trichiura[1,2]	18S rRNA	TTGAAACGACTTGCTCATCAACTT	CTGATTCTCCGTTAACCGTTGTC	CGATGGTACGCTACGTGCTTACCATGG
Strongyloides stercoralis[2]	Dispersed repetitive sequence	TCCAGAAAAGTCTTCACTCTCCAG	TGCGTTAGAATTTAGATATTATTGTTGCT	TCAGCTCCAGTTGAACAACAGCCTCCAA
Universal bacterial[1]	16S rRNA	TCCTACGGGAGGCAGCA	GGACTACCAGGGTATCTAATCCTG	CGTATTACCGCGGCTGCT
Lachnospiraceae (Lachno3 faecal indicator)[4]	16S rRNA	CAACGCGAAGAACCTTACCAAA	CCCAGAGTGCCCACCTTAAAT	CTCTGACCGGTCTTTAATCGGA
Bacteroides (HF183/BacR287 faecal indicator)[5]	16S rRNA	ATCATGAGTTCACATGTCCG	CTTCCTCTCAGAACCCCTATCC	CTAATGGAACGCATCCC
CrAssphage (CPQ_056 faecal indicator)[6]	orf00024	CAGAAGTACAAACTCCTAAAAAACGTAGAG	GATGACCAATAAACAAGCCATTAGC	AATAACGATTTACGTGATGTAAC

* Both assays required to be positive for *S. flexneri* serotype 6 to be detected, as per Liu *et al.* (2016)[3].

Table S4 (xlsx) – see Appendix 2. Lower limit of quantification (LLOQ) values for each target on standard qPCR and TAC.

Table S5 (xlsx) – see Appendix 2. Comparison of target copies per microlitre of undiluted genomic DNA quantified by TAC and standard qPCR for the spiked samples in different matrices.

Table S6 (xlsx) – see Appendix 2. Comparison of the effects of sample dilution on target detection by TAC in sewage samples.

Table S7 (xlsx) – see Appendix 2. Comparison of target copies per microlitre of undiluted genomic DNA quantified by TAC and standard qPCR for the human stool, animal scat, soil, and water samples from urban informal settlements of Suva, Fiji.

Supplementary references

1. Liu, J. *et al.* A Laboratory-Developed TaqMan Array Card for Simultaneous Detection of 19 Enteropathogens. *J. Clin. Microbiol.* **51**, 472–480 (2013).

2. Liu, J. *et al.* Optimization of Quantitative PCR Methods for Enteropathogen Detection. *PLOS ONE* **11**, e0158199 (2016).

3. Liu, J. *et al.* Use of quantitative molecular diagnostic methods to identify causes of diarrhoea in children: a reanalysis of the GEMS case-control study. *The Lancet* **388**, 1291–1301 (2016).

4. Feng, S., Bootsma, M. & McLellan, S. L. Human-Associated Lachnospiraceae Genetic Markers Improve Detection of Fecal Pollution Sources in Urban Waters. *Appl Env. Microbiol* **84**, e00309-18 (2018).

5. Green, H. C. *et al.* Improved HF183 Quantitative Real-Time PCR Assay for Characterization of Human Fecal Pollution in Ambient Surface Water Samples. *Appl Env. Microbiol* **80**, 3086–3094 (2014).

6. Stachler, E. *et al.* Quantitative CrAssphage PCR Assays for Human Fecal Pollution Measurement. *Environ. Sci. Technol.* **51**, 9146–9154 (2017).

Dataset S1. Information on the three plasmid inserts used for production of standard curves. Each plasmid contains synthetic primers and probes for each pathogen / indicator target.

Primers are underlined, with probes in bold.

Plasmid 1 insert:

ACCATCTACACATGACCCTCTATGAGT**AGTTAAAAGCTAACACTGTCAAA**GCTATAGGGGCGTTATGTGACCA
ACTTTCTCTCTTAATAGACGCCA**CTGACACGGGCACTCT**TTTTGTTTTCTAGCCCCCTCAGTTGCTTGCTGCGTT
CAT**CACAGAAGAGCAACTCCATCGC**AGAAGTGTGATGGCTAGCAAGCGCTGGATGCGCTTCCATGACAAGAG
CCAATGTTCAGATGGATGAG**TGGACAGGAGATCGCTGGGAGGGCGATCGCAATCT**GTAAATGATGATGGCG
TCTAAGTGTGAATGAAGATGGCGTCGAGATCAGGCTCTCGCCACCTACTTTGAACAAGCTGTGGCATGCTAC**C**
CTGGTTCATAGGTGGTACAGCAGCTGGTACATTGGTGGCACTAGTGTTTGAGATGGAGGGCCGGCAAACTG
GCTCTCGA**CAGTTCCAGTCCCACCACTT**CTTGTCCCACTGGTAACGAACTTTGAAGGTAATTTAGATATGGATA
ATCGA**CATTTTGACGATTTTTGGCTTGA**GTGAGAAATTTTATTTCTATGGTTTAGCAGTCAAATTGCATACCTTC
AACG**TTTGCCTCTTCAACAGT**ATAACACCATCTTTGCCCACTTTGGGACAAGGATTCTACCAGCTTTATAG**CAA**
TGGCGAATCCATCAGAATGATGAATGAGATTGCCGAATGACACCAGAAAAAGCGGCTAC**TCACTAAAGCGTT**
TTCTACCTTTAACCGAAGACATGCGCTTTTGTGCCATCATTTTCTAAGCA**CCTGGTGTCCATCCTGTTTC**CTATTT
GGAGCATTAGGTAATACCCCGCCGTGAAGGCAAAGT**CACACCCAAGAATAC**ATCTGGGTGAGCCGGTAGCCC
TTTTCCGCGTTCCTTGAC**CGCCTTTCCGATACCGTCTCTGCA**GCAATACCTCCGGATTCCGTGCCGCTAAAATCC
TTCTGTA**GAAGTTATTGATTCCGCCC**TTTTTCCTTTCGTCGTACGCTGGGTGCATCCTGACCTGTC**ACCACGGA**
ATAATCCCGCAGATCGGTAGCTCGTTATTGTTTGTC

Expected hits for all primers and probes in plasmid 1 (5′ to 3′):

Rotavirus F: 1-26
Rotavirus P: 28-50
Rotavirus R: 72-51

Adenovirus F: 73-95
Adenovirus P: 97-112
Adenovirus R: 131-113

Astrovirus F: 132-150
Astrovirus P: 152-173
Astrovirus R: 194-174

Norovirus GI F: 195-214
Norovirus GII F: 215-240
Norovirus GI P: 241-255
Norovirus GII P: 256-275
Norovirus GI R: 297-276
Norovirus GII R: 318-298

Sapovirus F(1): 319-339
Sapovirus F(2): 340-363
Sapovirus P(1): 364-384
Sapovirus P(2): 385-405
Sapovirus R: 424-406

Aeromonas F: 483-464
Aeromonas P: 444-463
Aeromonas R: 425-442

C. jejuni/coli F: 567-538
C. jejuni/coli P: 515-537
C. jejuni/coli R: 484-513

Campy F: 628-606
Campy P: 589-605
Campy R: 568-587

ETBF F: 629-654
ETBF P: 656-673
ETBF R: 696-674

H. pylori F: 697-716
H. pylori P: 718-737
H. pylori R: 757-738

C. difficile F: 824-801
C. difficile P: 781-800
C. difficile R: 758-779

P. shigelloides F: 825-841
P. shigelloides P: 843-857
P. shigelloides R: 876-858

Shig/EIEC F: 877-895
Shig/EIEC P: 897-920
Shig/EIEC R: 939-921

S. sonnei F: 940-959
S. sonnei P: 961-979
S. sonnei R: 999-980

S. flexneri periplasmic F: 1000-1018
S. flexneri periplasmic P: 1020-1039
S. flexneri periplasmic R: 1063-1040

Plasmid 2 insert:

CTCCTATCCGTGATTATAGTGCAG**TCCTTCTCACGATTAAAATC**AAATACAGTGAGTTGTGTGTGCCTTTCAAC
GCACGAATATCAACA**TTCTTCAGAACCGGGTTTTG**TCTCCGTCTGGATCAGGTTCTCGGGCAATTCGTTATTGG
C**AAGACAACAAAACCCACCGC**TGTCACCGTGGTCCAGTTTATCAAGACCTCAACGCCGATCACT**CAGCCTGCT**
CCAGAACACTATGTCGACTCTGACTTCGCGATTGTCCTCAGGCATTTCACT**TAGTGCATACTCATCATTTAAG**T
TGTTTATCAGGGGTGTCGTGCAATCAGATTAAGCAGCGATACAT**AAGACGCCTAAAGGATGCCC**GATGCTTG
CAGTTGTCCGAACTGGCGAAAGACTGTATCATA**TGGTTCTCATCTATTACAGACAGC**TCTATCGGCTTATGAA
GCAAAAGCTAAACCAGTAGAGTCTTCAAAATGAATCACTTGACTCTTCAAAA**TGGTCCTGAAAGCATGAATGA**
ACAACACATTTTACTGCTAACTTTGTTGTAATCCTGCCTGTTGTAATCCTGCTTGTACCGGGTTCCCACCGGATC
ACCAAG**CTTGGAGAGAAGAACCCT**TCATCATGCACCACAAGGTTGACTTCTCGACTGCAAAGACGTATGG**CTC**
TGCAATAGGTACTCCAGATAGTGGCACAGGGGATAATTTGTCCACATCGGTGTCTGTTATTAACCT**TTGCTGT**
GGATATACGAGGCTATCAGGCGCGTTTTGACCCATTGATCAGGATTTTTCTGGTGATAA**ATACTGGCGAGACT**
ATTTCAATAACGGCTATTTCCGCATGAGTGGTGCTTGCGCTTGCTC**CAGTCTGCGTCTGATTCCAA**CAGAAGT
AATGAGCGCAACGTGATTCACCAGCAGCAATACA**TGTCGGTTTCTCCTTCCAGG**CCGCCTTTCATGATGCCGG
GTTGTATTTATTAGATAAAGAACCAG**TGACATATCATTCAAGTTTCTGAC**AGACGGTAGGGTATTGGCCT

Expected hits for all primers and probes on plasmid 2 (5' to 3')

S. flexneri 6-O F: 1-23
S. flexneri 6-O P: 25-44
S. flexneri 6-O R: 66-45

S. flexneri 6-T3 F: 67-88
S. flexneri 6-T3 P: 90-109
S. flexneri 6-T3 R: 129-110

Salmonella F: 130-148
Salmonella P: 150-169
Salmonella R: 191-170

Typhi F: 251-230
Typhi P: 213-229
Typhi R: 192-211

aaiC F: 252-271
aaiC P: 273-294
aaiC R: 314-295

aggR F: 315-338
aggR P: 340-359
aggR R: 379-360

aatA F: 380-399
aatA P: 401-424
aatA R: 446-425

STh F: 447-470
STp F: 471-492
STh P: 493-510

STp P: 511-531
STh R: 575-552
STp R: 551-532

LT F: 576-593
LT P: 595-612
LT R: 633-613

Stx1 F: 634-657
Stx1 P: 659-677
Stx1 R: 702-678

Stx2 F: 703-726
Stx2 P: 728-746
Stx2 R: 766-747

eae F: 767-792
eae P: 794-814
eae R: 835-815

bfpA F: 836-852
bfpA P: 854-873
bfpA R: 893-874

Y. entero F: 894-913
Y. entero P: 915-934
Y. entero R: 951-935

Crypto F: 952-978
Crypto P: 980-1003
Crypto R: 1023-1004

Plasmid 3 insert:

AACACCAACGCACGCAGCT**AAGGCCGGATGACCACGA**CAGAAATCCAACTACGAGCTTTTATATTCCCTGCAG
CATGTCTGTTTG**CAAGTTCTGCTCACGCGCTTCTGG**TCTCTGGAATACGCGTGTGGGACGGCTCAGGACAACG
GTTC**CCCGCGGCGGTCCCTGCTAG**CCGGACACCGCTGGCAATCCCCCTGAAGTCCATAAACTCG**CCTTGTTCA**
GAACTTAAAGAGAAACAATCCTTGGTTGACATCGTTTATTGTCGTGGCATCCTAACTCAT**TCATTGAATGAAT**
TGGCCATTTTGTTATAATGAGCCGTCCGCCCTACGGAAACCTTGTTACGACTTCAT**CTTCCTCTAAATGATAAG**
ATTATCCAAAGTGTTCACCGGACCAGAATGACAGCAAACTCGTTGTTGC**ATCGTTTACCGACTTTAG**ACGTTTC
GGCAGTGGCTAGTATGCCACATAGTAAATTGCACACAAATG**TTGGCGGACAATTGCATGCGAT**ATGTTGGGC
TTGTTAGAAAGGCCTGTTTGTCGAACGGTACTTGCA**CTGTACTACGCATTGTATAC**GCAACATGTGCACGCTG
TTATTTGAAACGACTTGCTCATCAACTTT**CGATGGTACGCTACGTGCTTACCATGG**GACAACGGTTAACGGAG
AATCAGTCCAGAAAAGTCTTCACTCTCCAGC**TCAGCTCCAGTTGAACAACAGCCTCCAA**AGCAACAATAATAT
CTAAATTCTAACGCAGGACTACCAGGGTATCTAATCCTGC**CGTATTACCGCGGCTGCT**TGCTGCCTCCCGTAG
GACAACGCGAAGAACCTTACCAAAG**CTCTGACCGGTCTTTAATCGGA**ATTTAAGGTGGGCACTCTGGGCTTCC
TCTCAGAACCCCTATCCA**CTAATGGAACGCATCCC**CGGACATGTGAACTCATGATCAGAAGTACAAACTCCTA
AAAAACGTAGAGC**AATAACGATTTACGTGATGTAAC**GCTAATGGCTTGTTTATTGGTCATC

Expected hits for all primers and probes on plasmid 3 (5’ to 3’)

Cyclospora F: 60-38
Cyclospora P: 20-37
Cyclospora R: 1-18

Cystoisospora F: 61-84
Cystoisospora P: 86-109
Cystoisospora R: 110-129

Giardia F: 130-149
Giardia P: 151-170
Giardia R: 187-172

Entamoeba F: 256-235
Entamoeba P: 211-234
Entamoeba R: 188-209

E. histolytica F: 257-278
E. histolytica P: 280-301
E. histolytica R: 321-302

Blastocystis F: 391-370
Blastocystis P: 349-369
Blastocystis R: 322-347

Ancylostoma F: 392-414
Ancylostoma P: 416- 433
Ancylostoma R: 455-434

Ascaris F: 456-480
Ascaris P: 482-503
Ascaris R: 525-504

Necator F: 526-547
Necator P: 549-568
Necator R: 589-569

Trichuris F: 590-613
Trichuris P: 615-641
Trichuris R: 664-642

Strongy F: 665-688
Strongy P: 690-717
Strongy R: 746-718

16S F: 806-790
16S P: 772-789
16S R: 747-770

Lachno F: 807-828
Lachno P: 830-851
Lachno R: 872-852

Bacteroides F: 932-913
Bacteroides P: 896-912
Bacteroides R: 873-894

Crassphage F: 933-962
Crassphage P: 964-986
Crassphage R: 1011-987
